# Severe cardiac involvement with preserved truncated dystrophin expression in Becker muscular dystrophy by +1G>A *DMD* splice-site mutation: a case report

**DOI:** 10.1038/s10038-020-0788-9

**Published:** 2020-06-05

**Authors:** Ryouhei Komaki, Yasumasa Hashimoto, Madoka Mori-Yoshimura, Yasushi Oya, Hotake Takizawa, Narihiro Minami, Ichizo Nishino, Yoshitsugu Aoki, Yuji Takahashi

**Affiliations:** 1 Department of Neurology, National Center Hospital, National Center of Neurology and Psychiatry, Kodaira, Japan; 2 Department of Molecular Therapy, National Institute of Neuroscience, National Center of Neurology and Psychiatry, Kodaira, Japan; 3 Department of Neuromuscular Research, National Institute of Neuroscience, National Center of Neurology and Psychiatry, Kodaira, Japan; 4 Department of Genome Medicine Development, Medical Genome Center, National Center of Neurology and Psychiatry, Kodaira, Japan

**Keywords:** Genetic testing, Molecular biology

## Abstract

Becker muscular dystrophy (BMD) is caused by specific mutations in the *DMD* gene that causes progressive muscle weakness and primarily affects skeletal and cardiac muscle. Although cardiac involvement is a significant cause of mortality in BMD, the genetic–phenotype correlation for skeletal and cardiac muscles has not been elucidated. Here, we described a 39-year-old man with BMD, who presented with subtle skeletal muscle weakness in the right leg in his 20s and underwent left ventricular restoration for severe dilated cardiomyopathy at the age of 29. He had difficulty climbing stairs after the age of 35. Neither duplication nor deletion of exons was detected by multiplex ligation-dependent probe amplification. A hemizygous c.264 + 1G>A mutation in intron 4 of the *DMD* was identified by next-generation sequencing. Furthermore, exon 4 skipping of the *DMD* was confirmed in both skeletal and cardiac muscles evaluated by reverse transcriptase PCR. Endomyocardial and skeletal muscle biopsies revealed dystrophic pathology characterized by muscle fiber atrophy and hypertrophy with a mild degree of interstitial fibrosis. Interestingly, dystrophin immunohistochemistry demonstrated patchy and faint staining of the skeletal muscle membranes but almost normal staining of the cardiac muscle membranes. Western blot analysis revealed a decreased amount of truncated dystrophin in skeletal muscle but surprisingly almost normal amount in cardiac muscle. This case indicates that BMD patients may have severe cardiac dysfunction despite preserved cardiac truncated dystrophin expression.

## Introduction

Dystrophinopathies, including Duchenne muscular dystrophy (DMD), Becker muscular dystrophy (BMD), and X-linked dilated cardiomyopathy (XLDCM), are genetic muscle dystrophies of X-linked inheritance caused by mutations in the *DMD* gene on the X chromosome [1].

Individuals with DMD and BMD classically experience muscle weakness as an initial symptom before the manifestation of cardiac symptoms [2]. Out-of-frame deletions producing insufficient and nonfunctional proteins causes severe clinical phenotypes classified as DMD [2]. In contrast, BMD, typically resulted from in-frame deletion, is diagnosed when the patient’s ambulatory ability is comparatively preserved. Although the reading-frame rule accounts for over 90% of dystrophinopathies [3], exceptions to this rule exist. Clinical characteristics and especially the degree of cardiac involvement vary from asymptomatic to severely symptomatic in young BMD patients. The severity and age of onset of cardiac involvement do not show any correlation with the degree of skeletal muscle involvement [4]. Of note, some BMD patients present with severe cardiac dysfunction, including arrhythmias and dilated cardiomyopathy (DCM) preceding the development of skeletal muscle weakness. Moreover, cardiovascular complications are a leading cause of disease-related mortality in BMD [5]. Thus, understanding the correlations and mechanism of cardiac involvement is essential for the development of novel therapeutics.

There are various types of gene mutations in BMD; exon deletions and duplications are particularly common major mutation types, which can be detected by multiplex ligation-dependent probe amplification (MLPA). Approximately 30% of patients have other mutations, namely, splice-site mutations, nonsense mutations, micro-deletions, or insertion mutations [6]. The pathophysiological mechanisms underlying these minimal change mutations, including splice-site mutations, remain elusive.

Here, we describe a case of BMD caused by a splice-site mutation in intron 4 of the *DMD* gene and compare the clinical characteristics, pathological changes, *DMD* mRNA expression, and dystrophin protein content in skeletal and cardiac muscles to elucidate the mechanisms underlying the discrepancy between skeletal and cardiac dysfunction.

## Materials and methods

### *DMD* DNA analysis

After providing informed consent, genomic DNA from blood lymphocytes of this patient was used for next-generation sequencing using Ion PGM sequencer covering the *DMD* gene previously described [7], and the result was confirmed by Sanger sequencing.

### *DMD* mRNA analysis

After providing informed consent, the patient underwent an endomyocardial biopsy at the age of 29 and a skeletal muscle biopsy from the left biceps brachii at the age of 39. Total mRNA was extracted from the frozen skeletal and cardiac muscle biopsies, and complementary DNA was synthesized using random hexanucleotide primers following the manufacturer’s protocol. Reverse transcription PCR was performed utilizing intra-exonic primers for *DMD* exons 3 and 6 designed with the Leiden Muscular Dystrophy pages (https://www.dmd.nl/); it was followed by direct sequencing. The primer sequences (5′–3′) were as follows: forward, GGGAAGCAGCATATTGAGAA; reverse, ATGAGAGCATTCAAAGCCAG.

The PCR reactions utilized Taq polymerase, 0.5 M of each primer, and 0.8 μM dNTP. The amplification conditions were as follows: denaturation at 94 °C for 60 s, annealing at 66 °C for 60 s, and extension at 72 °C for 30 s for 35 cycles. Eighteen microliters of the reaction products were analyzed on MultiNA (Shimadzu, Kyoto, Japan), the resulting PCR bands were extracted using a gel extraction kit (Qiagen), and direct sequencing of the PCR products was performed by the Eurofins (Tokyo, Japan).

### Protein extraction and immunoblotting analysis

Total protein was extracted from the patient’s cardiac and skeletal muscle specimens using RIPA buffer containing protease inhibitors (Roche, Indianapolis, IN, USA). The lysates were sonicated on ice and centrifuged at 20,000 × rpm for 30 min at 4 °C. The supernatant was collected, and protein concentrations were determined employing a BCA protein assay kit (Thermo Fisher Scientific). After mixing with NuPAGE LDS Sample Buffer (Thermo Fisher Scientific), cell lysates were denatured at 70 °C for 10 min, electrophoresed utilizing a NuPAGE Novex Tris-acetate gel 3–8% (Invitrogen) at 150 V for 70 min, and then transferred to PVDF membranes. The membranes were incubated with primary antibodies, followed by incubation with a secondary antibody using the iBind Flex Western Device (Thermo Fisher Scientific). The following primary antibodies were utilized: rabbit antidystrophin (1:500, Abcam, UK; ab15277), mouse antiactinin (1:1000, Sigma-Aldrich, UK; A7811) and antitubulin antibody (1:1000, Sigma-Aldrich, UK; T6199); Histofine Simple Stain MAX-PO (1:100, NICHIREI BIOSCIENCE INC., Tokyo, Japan; 424151) was used as a secondary antibody. Proteins were detected utilizing the ECL Prime Western Blotting Detection Reagent (GE Healthcare, UK; RPN2232), and a ChemiDoc MP Imaging System (Bio-Rad, Hercules, CA, USA). The data were analyzed with Image Lab 6.0 (Bio-Rad).

## Results

### Case presentation

**Fig. 1:**
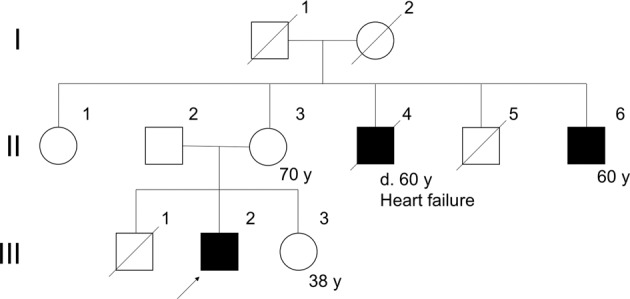
Pedigree of the patient’s family. The blackened squares denote affected patients

A 39-year-old man presented with gait disturbance and cardiac dysfunction. His maternal uncle died of heart failure developing after the development of gait disturbance and hyper-creatine kinase (CK)-emia since age 20 (Fig. 1, Case II-4). Another of his maternal uncle suffered from lower limb weakness and hyper-CK-emia developing after the age of 20 (Fig. 1, Case II-6). In both cases, the muscular dystrophy diagnosis was suspected but not genetically confirmed. The patient had two siblings who did not have neurodegenerative disorders.

**Fig. 2:**
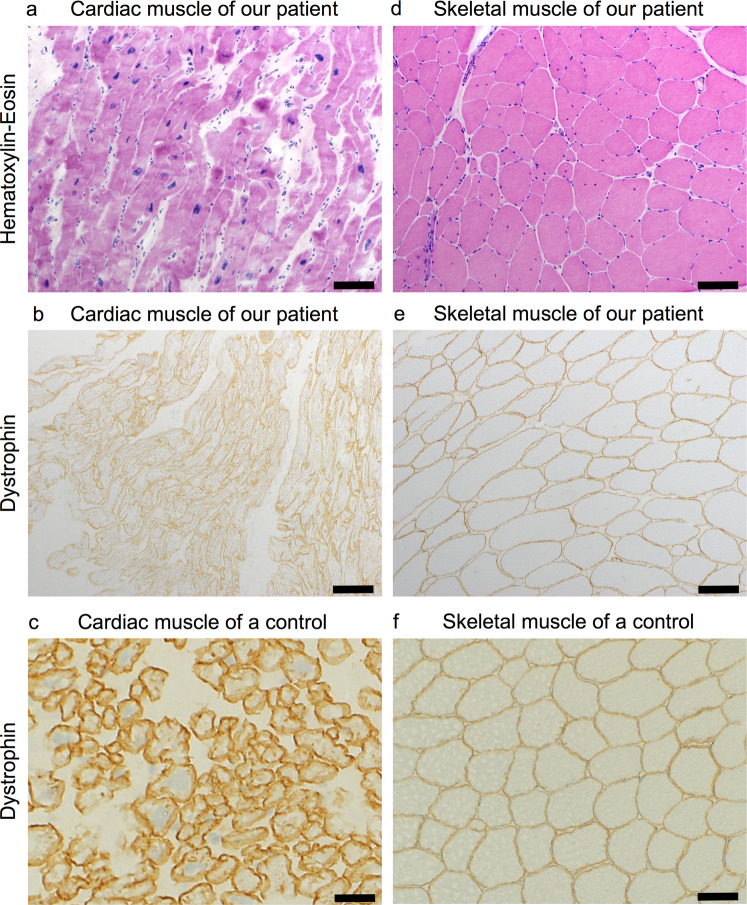
Muscle pathological analyses of the patient. Hematoxylin–eosin staining of (**a**) cardiac and (**d**) skeletal muscle tissue from the patient. Bar, 100 μm. Immunohistochemistry of Dys2 expression in cardiac muscle from our patient (**b**), and cardiac muscle from a control individual (**c**). Bar, 100 μm. Immunohistochemistry of Dys2 expression in skeletal muscle from our patient (**e**), and skeletal muscle from a control individual (**f**). Bar, 100 μm

His growth and developments were intact. He could not run rapidly compared with his peers in childhood and at that time noticed calf enlargements. He played table tennis in his junior high school days but did not do regular exercise since the age of 15. His occupation was an office worker to do desk work. The patient felt a slight weakness in the right leg in his 20s. At the age of 29, he experienced mild dyspnea on exertion due to DCM diagnosed by cardiac ultrasound. It showed a 22% ejection fraction, diffuse hypokinesis of the left ventricle wall, diastolic/systolic ventricle size of 76/68 mm, respectively, severe mitral valve regurgitation, and mild trigeminal valve regurgitation. The serum CK level was elevated (4283 U/mL; normal range 62–287 U/L). The patient underwent a left ventricular restoration, mitral valvuloplasty, tricuspid valvuloplasty, and cardiac resynchronization therapy, proceeding to cardiac rehabilitation. Endomyocardial biopsy from left ventricle showed a fiber size variation, marked fibrosis, and minimal lymphocyte infiltration, suggesting muscular dystrophy (Fig. 2a). The immunohistochemical analysis did not reveal a deficit of dystrophin protein (Fig. 2b). Treatment started with cardiac protective medications, including enalapril, spironolactone, and carbezirol. The patient had gradually developed difficulties climbing stairs since the age of 36. He was admitted to our hospital for further evaluations at the age of 39.

On physical examination, his heart sounds were intact, and no heart murmur was observed. His muscle strength determined with the Medical Research Council grading revealed the following distributions (right/left): neck flexor 4, truncal flexion 4, proximal upper extremities 4/4, distal upper extremities 5/5, hip abduction & adduction 3/3, knee extensor & flexors 4/4, and lower distal extremities 5/5. His grip strength was 21.5/22.5 kg.

Slight atrophy of the bilateral proximal quadriceps and calf hypertrophy were observed. The patient had mild lordosis. He did not require ambulatory aids to walk. Other neurologic examinations, including assessments of cognitive function, cranial nerves, reflexes, and sensory, corporative, and autonomic functions were all intact.

Laboratory examinations showed an elevated serum CK level (2845 U/L) and brain natriuretic peptide level (59.1 pg/mL; normal range 5.79–18.4 pg/mL). Chest X-ray did not reveal a cardiac enlargement (cardiothoracic ratio 48%) or any lung shadows. Electrocardiogram showed normal sinus rhythm and T-wave inversion on V5 and V6. No arrhythmia was detected. Cardiac ultrasound demonstrated cardiac dysfunction, including a 24% ejection fraction, left posterior wall akinesis, a diastolic/systolic ventricle size of 67/60 mm, respectively, a mild mitral valve regurgitation, and a trivial trigeminal valve regurgitation. Needle electromyography demonstrated positive sharp waves, fibrillation potentials, and low amplitude motor unit potentials in both right biceps brachii and vastus lateralis muscles, indicating a myogenic change.

Muscle biopsy of left biceps brachii revealed a moderate to marked variation in fiber size, a few necrotic and regenerating fibers, and an increased number of fibers with internal nuclei (51%) (Fig. 2d). Immunohistochemical analysis showed a patchy and faint reaction of the dystrophin sarcolemma (using N-terminal) (Fig. 2e).

**Fig. 3:**
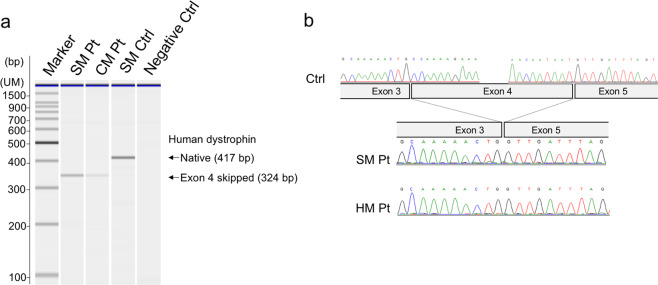
mRNA analysis using reverse transcriptase PCR. Exon 4 skipping was analyzed by reverse transcriptase PCR. **a** Densitometry analysis of exon 4 skipping as represented by a microchip-based capillary electrophoresis system is shown. UM upper marker dye. **b** Sequencing of skeletal muscle and cardiomyocyte mRNA from the patient revealed skipping of exon 4. SM Pt skeletal muscle from the patient; CM Pt cardiomyocytes from the patient; SM Ctrl skeletal muscle from a healthy control

**Fig. 4:**
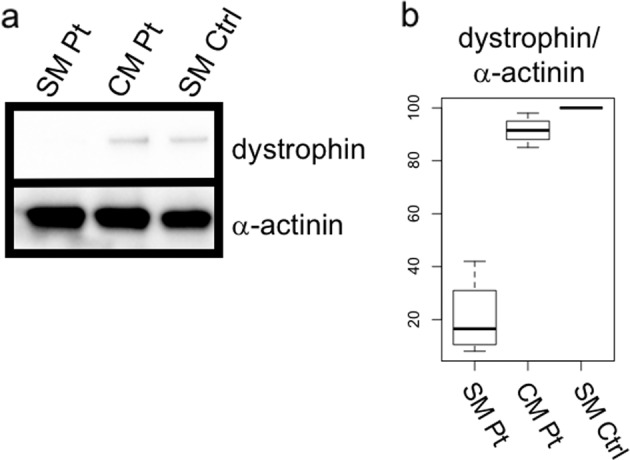
Western blotting analysis of dystrophin. The dystrophin level was decreased to 20.90% (±15.15%) in skeletal muscle and 91.53% (±5.39%) in cardiac muscle compared with that in skeletal muscle from a healthy control. The experiment was repeated three times (**a**, **b**)

MLPA demonstrated no duplication and deletion mutations. Next-generation sequencing revealed a hemizygous c.264 + 1G>A mutation in intron 4. The result was confirmed by sanger sequencing. Based on the clinical and genetic evidence [8], the patient was diagnosed with BMD. To further analyze the actual mechanisms of the +1G>A splicing mutation in intron 4, mRNA analysis (reverse transcriptase PCR) was performed. It showed exon 4 skipping in both skeletal and cardiac muscles (Fig. 3). Western blot analyses revealed that the truncated dystrophin level was decreased to 20.90% (±15.15%) in skeletal muscle and 91.53% (±5.39%) in cardiac muscle compared with that in skeletal muscle from a healthy control (Fig. 4).

## Discussion

We described the case of a patient with BMD caused by c.264 + 1G>A mutation in intron 4 donor splice site of the *DMD* gene. His clinical characteristics included a severer cardiac phenotype than skeletal muscular involvement. Although we failed to detect the splice-site mutation by MLPA, we identified the mutation, which induced exon 4 skipping of the *DMD*, by mRNA analysis. Surprisingly, western blot analyses showed that the expression level of truncated dystrophin was almost normal in cardiac muscle but reduced in skeletal muscle. This case indicates that even when the truncated dystrophin is preserved in cardiomyocytes, patients with BMD could have severe cardiac dysfunction.

Splice-site mutations of the *DMD* gene were commonly detected in +1 point mutations in introns [3]. It is essential for clinicians to know that MLPA sometimes fails to detect splice-site mutations [6]. In addition to destructing normal splice sites, these mutations construct new splice sites, demolish, and produce splicing regulatory elements, and occasionally activate pseudoexons [9]. These processes result in various phenomena, including exon skipping, cryptic splice-site activation, partial exon loss, or intron retention [10]. Consequently, an abnormal dystrophin protein is synthesized. Important mechanisms based on mutations were different depending on the mutation sites. The expected phenotype was different between analyses; for example, a computer-based analysis suggested that exon skipping might occur, if the mutation was localized in exons longer than 170 bp [11], and, according to the Human Splicing Finder (http://www.umd.be/HSF3/technicaltips.html), a cryptic splice-site activation might add 19 amino acids after exon 4. We detected for the first time that this +1G>A mutation in intron 4 caused skipping of exon 4. We also emphasized that the actual genetic phenomenon occurring in patients should be determined by genetic experiments such as mRNA analysis.

The genetic–phenotype correlation in dystrophinopathies has been previously hypothesized, but the available information is limited [12]. Genes, including muscle promoter to muscle exon 1, exons 2–8 region coding the actin-binding domain [13, 14], exons 45–55, cysteine-rich domain, and carboxyl terminus [4], have been associated with a severe phenotype of cardiac involvement in BMD and XLDCM.

Other plausible mechanisms of severer cardiac dysfunctions than skeletal muscular manifestation in our case include following. First, different transcriptional regulation between skeletal and cardiac muscles, resulting in that different isoforms such as the full-length brain (B) and cerebellar Purkinje (P) dystrophin, compensate a lack of full-length muscle dystrophin in skeletal muscles not in cardiac muscles [15]. Second, enterovirus and coxsackievirus infections cause severe cardiac manifestations by enteroviral protease 2A cleaving the dystrophin protein [16]. However, these theories were not fully compatible with our case because the amount of dystrophin protein in cardiac muscle was preserved. Other hypotheses include conformational change of dystrophin and impairments of interaction between dystrophin and its binding proteins. Duplication, involving exons 2–7, results in a deficiency of cardiac dystrophin protein, accompanied by a reduction of sarcolemma dystrophin-associated proteins (beta-dystroglycan, alpha-sarcoglycan, and syntrophin) [17]. In addition, the actin-binding domain may be more crucial for cardiac muscle than for skeletal muscle for the avoidance of mechanical stress due to deficient cytoskeletal components [18]. The mutation identified in the present study might have resulted in a conformational change of truncated dystrophin and deterioration of its interactions with other dystrophin-associated proteins and actin, although domains which binds with these dystrophin-associated proteins and actin were not affected. Finally, excess exercise may have a negative influence on cardiac muscles because it causes extensive cardiac muscle inflammation and fibrosis without decrease of dystrophin expression via activations of p38 mitogen-activated protein kinase, extracellular signal-regulated kinase 1/2, and calcineurin [19].

There are two previous case reports of BMD caused by exon 4 deletion. One patient had severe DCM compared with skeletal muscle weakness developing during his 30s and was diagnosed at 47-year old [20]. Other four siblings who had the deletion of exon 4 of *DMD* showed hyper-CK-emia and severe mental retardation without apparent muscle weakness and cardiac involvement at least until 33 years old [21]. Since the lack of muscular dysfunction in the latter case is not line with the findings in our case, other mechanisms, including protein–protein interactions, should be considered to understand the exact pathophysiology.

Our report has several limitations. First, we could not compare the protein expression in cardiomyocytes from our patient with that in cardiomyocytes from a healthy control. Alternatively, we compared the dystrophin expression level in cardiac muscles derived from BMD and Pompe disease and found that the expression of dystrophin was quite similar in those cardiac muscles (Supplementary Figure). Furthermore, dystrophin expression level in skeletal and cardiac muscles are reported to be quite similar in both human and mouse [22, 23]. Thus, we could not conclude whether dystrophin protein expression was actually decreased in cardiac muscle. However, the amount of dystrophin protein in cardiac muscle in our patient was at least not markedly reduced. This result indicated that the severer cardiac phenotype than skeletal muscular manifestation was caused by other mechanisms. Second, seemingly slight expression of *DMD* mRNA in cardiac muscles may be explained by multiexons skipping beyond exon 3 and 6 or intron retention which could be missed by reverse transcriptase PCR. Third, the expression of other dystrophin-associated proteins was not fully evaluated.

Our case has implications for future gene therapy targeting cardiomyopathy associated with dystrophinopathies. In addition to conventional therapies, including beta-blockers, angiotensin-converting enzyme inhibitors, and corticosteroids, many genomic treatment approaches, including exon-skipping therapy, are being developed. Currently, the indications are restricted to patients genetically diagnosed with DMD caused by an exon deletion, which targets exon 44, 45, 51, or 53 [23]. A previous report demonstrated that a deletion of exons 3–9 was associated with an asymptomatic phenotype at the age of 27, indicating that multiexon skipping therapy might become a therapeutic option for patients not only with DMD but also with BMD accompanied with severe cardiac dysfunction [24].

Further studies are needed to elucidate the detailed mechanism of other mutations, especially ones associated with severe phenotypes, which cannot be explained by the reading-frame rule. These mutations may be a candidate for genetic treatments and may provide a detailed understanding of the disease pathogenesis.

In conclusion, we described the case of a patient with BMD caused by +1G>A mutation in intron 4 of the *DMD* gene. His cardiac dysfunction was severer than skeletal muscle dysfunction, even though immunostaining and western blot analysis revealed that the truncated dystrophin protein was preserved in his cardiomyocytes. Clinicians should be aware that patients with BMD may show a severe cardiac dysfunction, even when the dystrophin protein in cardiomyocytes is relatively preserved.

## Supplementary information

Supplementary Figure
