# Supplementary material for: Severe cardiac involvement with preserved truncated dystrophin expression in Becker muscular dystrophy by +1G>A DMD splice-site mutation: a case report
Source: J Hum Genet. 2020 Jun 5;65(10):903–9. doi: 10.1038/s10038-020-0788-9 (PMC7449875; doi:10.1038/s10038-020-0788-9)
Supplement: Supplementary file 1 — Supplementary Figure [file 10038_2020_788_MOESM1_ESM.pptx]

## Slide 1
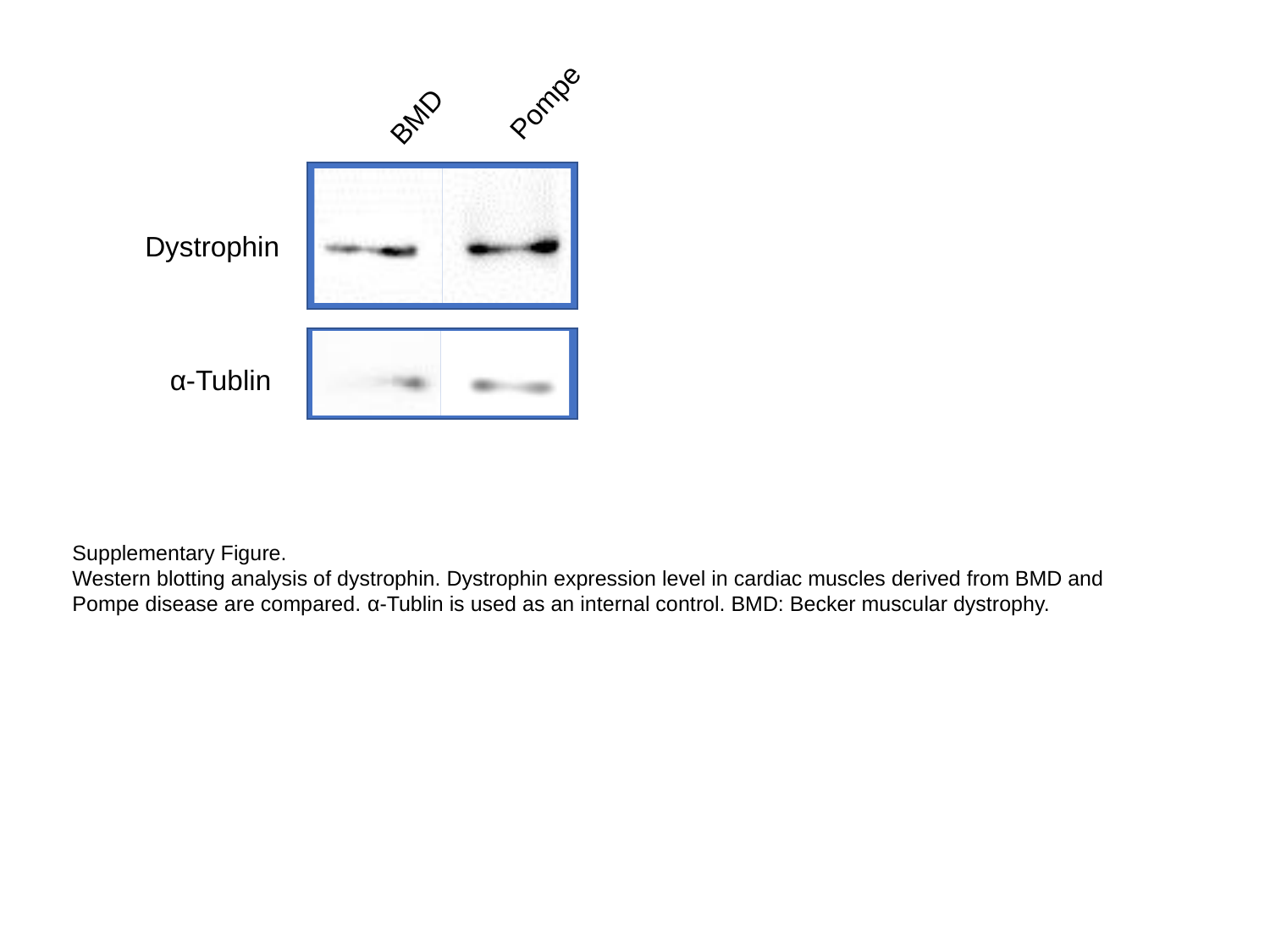

Pompe
BMD
Dystrophin
α-Tublin
Supplementary Figure.
Western blotting analysis of dystrophin. Dystrophin expression level in cardiac muscles derived from BMD and Pompe disease are compared. α-Tublin is used as an internal control. BMD: Becker muscular dystrophy.
